# Dietary restriction during the treatment of cancer: results of a systematic scoping review

**DOI:** 10.1186/s12885-019-5931-7

**Published:** 2019-08-15

**Authors:** Ellie Shingler, Rachel Perry, Alexandra Mitchell, Clare England, Claire Perks, Georgia Herbert, Andy Ness, Charlotte Atkinson

**Affiliations:** NIHR Bristol BRC Nutrition Theme, Level 3, University Hospitals Bristol Education Centre, Upper Maudlin Street, Bristol, BS2 8AE UK

## Abstract

**Background:**

Diets that restrict energy or macronutrient intake (e.g. fasting/ketogenic diets (KDs)) may selectively protect non-tumour cells during cancer treatment. Previous reviews have focused on a subset of dietary restrictions (DR) or have not performed systematic searches. We conducted a systematic scoping review of DR at the time of cancer treatment.

**Methods:**

MEDLINE, Embase, CINAHL, AMED and Web of Science databases were searched for studies of adults undergoing DR alongside treatment for cancer. Search results were screened against inclusion/exclusion criteria. Data from included studies were extracted by two independent reviewers. Results were summarised narratively.

**Results:**

Twenty-three independent studies (34 articles), with small sample sizes, met the inclusion criteria. Four categories were identified: KDs (10 studies), fasting (4 studies), protein restriction (5 studies) and combined interventions (4 studies). Diets were tolerated well, however adherence was variable, particularly for KDs. Biomarker analysis in KDs and fasting resulted in the expected increase in ketones or reduction in insulin-like growth factors, respectively, however they did not reduce glucose.

**Conclusions:**

Future research with adequately powered studies is required to test the effects of each DR intervention on treatment toxicities and outcomes. Further research into improving adherence to DR may improve the feasibility of larger trials.

**Electronic supplementary material:**

The online version of this article (10.1186/s12885-019-5931-7) contains supplementary material, which is available to authorized users.

## Background

Pre-clinical studies in model organisms have identified the potential protective effect of restricting overall energy intake or specific macronutrient intake on resistance to stress in these models. This has led to a growing interest in the use of restrictive diets to potentially attenuate the cytotoxic effects of cancer treatments such as chemotherapy and radiotherapy [1]. Examples of diets of interest include fasting, which restricts overall energy intake, and ketogenic diets, which restrict energy intake from carbohydrate sources. Collectively these diets can be referred to as dietary restriction (DR) [2].

### Cellular metabolism in cancer

When nutrients are not available, non-tumour cells are able to alter their cell signalling processes, withdrawing energy from growth/reproduction in order to conserve their energy for maintenance/repair. This leads to increased cellular protection [3]. This process is partially mediated by a reduction in growth factors, specifically insulin-like growth factor-1 (IGF-1) [4]. A reduction in IGF-1 reduces the activation of the Ras/MAPK and P13K/Akt pathways that promote expression of genes involved in proliferation, growth, survival and increased protein synthesis via mTOR.

Conversely, cancer is a disease associated with dysregulated metabolism [5]. One of the hallmarks of cancer is the ability of tumour cells to continue to grow in the absence of growth factors, such as when nutrients are scarce [6]. Mutated tumour cells evade these signals due to gain-of-function mutations in oncogenes (Ras, Akt, mTOR), which results in proliferation pathways continually being active, even in the absence of growth signals [3]. Therefore, tumour cells do not respond to nutrient deprivation in the same way as healthy cells and continue to proliferate, even when nutrients are scarce.

Furthermore, tumour cells are known to rely on glycolysis for energy production, the phenomenon of fermenting glucose to form lactic acid, rather than mitochondrial oxidative phosphorylation, even in the presence of oxygen [7]. This process has been coined the Warburg effect and can be thought of as a trade-off of “catabolic efficiency for anabolic utility” as the energy produced by the fermentation of glucose can be used for biosynthesis required for daughter cells during proliferation [8, 9]. This metabolic dysregulation is seen in nearly all tumour cells and may put these cells under increased pressure when glucose availability is low, requiring cells to switch from glucose metabolism to ketone metabolism and fatty acid oxidation [10].

This difference in reaction to nutrient scarcity between healthy and tumour cells is termed differential stress resistance and may render tumour cells more susceptible to the effects of chemotherapy while at the same time helping to protect healthy cells against the toxic effect of chemotherapy [1]. It is thought that through mechanisms such as decreased growth signalling for healthy cells and the lack of metabolic adaptability found in tumour cells, DR may lead to the increased vulnerability of tumour cells to treatment. Therapeutic regimes that take advantage of this differential stress resistance are therefore a potential tool in the treatment of cancer.

### Dietary restriction (DR)

Methods of DR such as fasting, carbohydrate restriction or protein restriction are dietary strategies which aim to exploit this difference in energy metabolism between healthy and tumour cells [11].

Chronic energy restriction and fasting lead to reduced blood glucose and IGF-1 and increased ketones [12]. However, chronic energy restriction may not be suitable for patients undergoing treatment with chemotherapy or radiotherapy due to the increased risk of cachexia and sarcopenia [3, 10]. Short term fasting (for example complete energy restriction lasting up to 4 days) at the time of chemotherapy has therefore been suggested as a potential therapy without the risks of chronic energy restriction [2]. More recently, a fasting mimicking diet has been created that mimics the physiological effect of fasting without having to reduce daily energy intake below 725 kcal. This diet aims to overcome some of the difficulties of short term, water only fasting, such as issues with adherence, adverse effects and malnourishment [12].

As well as energy restriction, the composition of restricted diets may also be of importance. Ketogenic diets (KDs) are high in fat with restricted carbohydrate intake. For example, the 4:1 KD comprises fats in a 4:1 ratio to carbohydrates, whilst also limiting protein intake, so that approximately 90% of calories are derived from fat [13]. KDs simulate many of the physiological responses of energy restriction such as a reduction in blood glucose and IGF-1 coupled with an increase in ketones [10, 11].

Protein restriction is another form of macronutrient restriction of interest. Epidemiological research has found that people following energy unrestricted plant-based diets, with reduced protein, have lower IGF-1 concentrations than those on long-term severe calorie restriction with adequate protein. This suggests that protein restriction may be another therapeutic strategy [14]. Protein restricted diets aim to reduce intake of total protein or of specific essential amino acids. Methionine is of particular interest, as an amino acid that has been recognised to have an important role in cellular metabolism. It is required for protein synthesis and DNA methylation required in cell growth/proliferation [15].

### Previous reviews

Reviews on DR that have been published to date have focused on subsets of DR studies and not all have been systematic in their search criteria.

Previous systematic reviews have been conducted in fasting [16] and KDs [17]. The review of fasting included studies on the effects of chemotherapy and studies on tumour progression, without chemotherapy. Authors concluded that fasting was seen to reduce chemotherapy side effects and suppress tumour progression. They also concluded that a 24 h fast may not be long enough for the protective effects of fasting to apply, due to two human studies which found less toxicities in 72 h fasts when compared to 24 h fasts [17, 18]. The review of KDs was not specifically in populations receiving active treatment for cancer [17]. Authors report inconclusive evidence for changes in nutritional status and adverse events as well as low adherence to KDs. No systematic reviews have been conducted on other forms of DR during treatment for cancer e.g. fasting mimicking diets or protein restriction.

In addition to the systematic reviews, two perspective reviews describing the rationale behind fasting and fasting mimetics at the time of chemotherapy have also been identified [3, 10]. These reviews describe how the findings in simple organism and animal models provide a rationale behind the use of some forms of DR and provide an overview of previous [3] and ongoing [10] DR trials. These reviews, however, do not describe a systematic search of the literature, and additional studies on DR in humans, not included in these reviews, have been identified.

Unlike a systematic review and meta-analysis, scoping reviews have a broader scope and provide a description of current evidence, regardless of quality [18]. This allows research on emerging fields, such as DR during treatment for cancer, which may not yet have results from many randomised controlled trials, to be presented and summarised in a systematic way. As such, a comprehensive systematic scoping review to identify studies in humans looking at the different types of DR at the time of cancer treatment is warranted. This review will provide a clear overview of research in this area to date and identify future research priorities.

### Aims and objectives

The aim of this scoping review is to summarise the research on the effects of dietary restriction on cancer treatment induced toxicities and outcomes in adult patients undergoing treatment for any malignancy.

The primary objective is to identify and characterise the research that has been conducted to date on dietary restriction as an adjuvant therapy in the treatment of cancer in adults with cancer. The secondary objective is to explore the acceptability of dietary restrictions in the samples identified through the search.

## Methods

A scoping review protocol was developed and made publicly available prior to commencement of this review [19].

### Inclusion criteria

Inclusion criteria were defined in terms of Population, Concept and Context [20].

#### Population

Adult participants undergoing some form of dietary restriction as an adjuvant treatment for any type of cancer.

#### Concept

Any form of dietary restriction studies which assessed:

i) The safety or feasibility of the interventions and/or
ii) The effect of the interventions on outcomes such as chemotherapy toxicities, clinical outcomes or cancer biomarkers. Examples of the dietary restriction forms of interest are short/long term fasts, intermittent fasts, fasting mimicking diets, ketogenic diets or protein restriction diets.

#### Context

Any cancer care setting. The intervention could be delivered in combination with any standard treatment for cancer e.g. chemotherapy, radiotherapy or immunotherapy.

### Exclusion criteria

Studies of animal models or model organisms were not included in this review. Although not specified in our original protocol, as we were interested in diets that initiate the metabolic changes associated with differential stress resistance and not diets that altered macronutrient composition without aiming to induce such changes, low fat diets which solely aimed to reduce weight in cancer populations were also excluded.

### Types of sources

All forms of quantitative and qualitative primary research were included, as were systematic reviews and meta-analyses. As dietary restriction is an emerging field, observational studies, case reports and conference abstracts were included in addition to trials. There were no limitations on date or language of publication.

### Search strategy

The following databases were searched for relevant articles on the 4th January 2018:

1. MEDLINE, Embase, AMED (via OVID)
2. CINAHL
3. Web of Science

An example of the search strategy used in MEDLINE is shown in Additional file 1 The search terms were updated for each database, in accordance with their specific requirements.

In addition to the database searches, the reference lists of all included articles were hand searched for additional studies alongside relevant systematic reviews. The ClinicalTrials.gov website was searched to identify any trials currently taking place which have not yet been completed or published. As an addition to the original protocol, the ISRCTN database was also searched for planned or ongoing trials. Finally, the first ten pages of google scholar were hand searched for any additional articles.

The results from the database searches were imported into an Endnote library and duplicates were removed during the data screening process.

### Selection of studies

Titles and abstracts of the search results were screened independently by two reviewers from a team of five researchers. Any discrepancies were discussed with a third reviewer for resolution, if required. Articles identified for potential inclusion were retrieved in full for further screening against the inclusion/exclusion criteria. Full texts which met the inclusion criteria underwent data extraction.

### Data extraction

Data charting forms were used to extract relevant data from the included studies. Charting forms were completed by two reviewers independently, then compared for accuracy. Extracted data included:

- Publication Information – Paper title, author details, publication type, study type, year of study
- Aims/purpose of the research
- Study population – sample size, demographics (age, sex, ethnicity), cancer site and staging, inclusion/exclusion criteria, withdrawals and exclusions
- Intervention type and design – Study design, intervention description (including type, timing and duration of dietary restriction)
- Key findings – Outcomes reported, and the outcome measures used, adverse events, adherence rates, acceptability and tolerability.

## Results

**Fig. 1:**
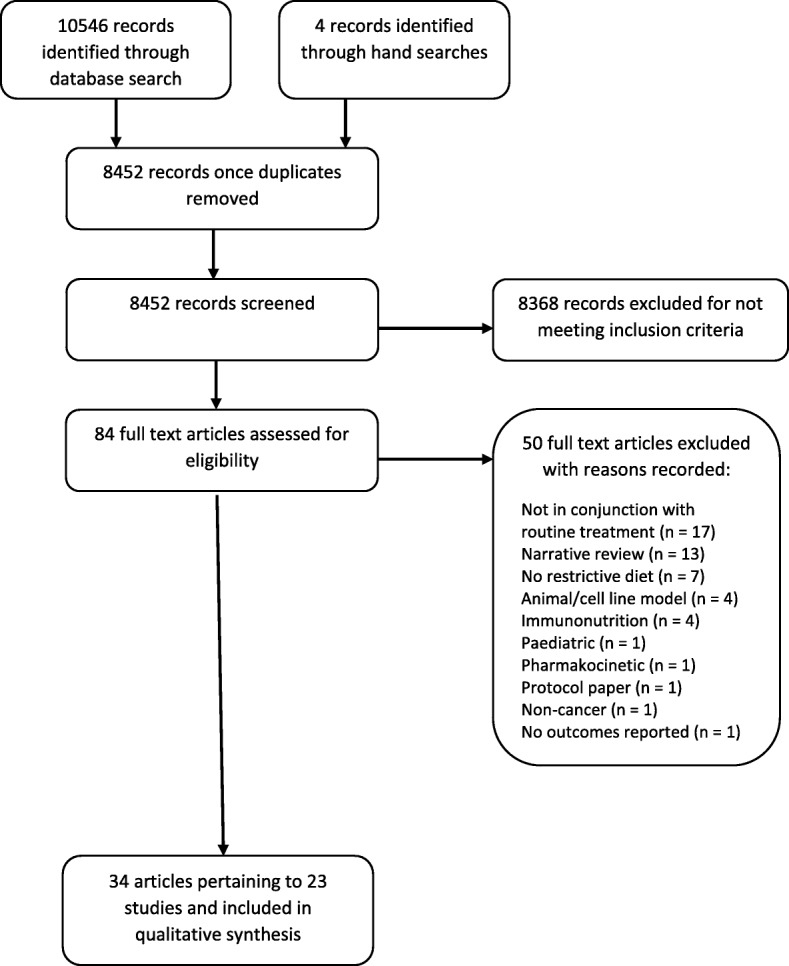
Inclusion flowchart

The inclusion flowchart for the review can be seen in Fig. 1.

The database search retrieved 8448 texts for screening and 4 additional manuscripts were identified through hand searches. Title/abstract screening identified 84 texts for full text screening. Fifty were excluded, with reasons recorded in Fig. 1. Thirty-four full texts which pertained to 23 studies in total, were identified for inclusion in the review and underwent data extraction.

### Characteristics of included studies

The 23 included studies were published between 2007 and 2017 and included a total sample size of 990 (range 1–596 in the observational studies and range 6–73 in the interventional studies). Four categories of interventions were identified: KDs, fasting, protein restriction and combined interventions. The majority of studies were of KD (*n* = 10), followed by protein restriction (*n* = 5), fasting (*n* = 4), and combined interventions (*n* = 4). The outcomes reported were varied, ranging from withdrawal rates, treatment side effects (both standard treatment and/or intervention effects) and biological markers. Results were therefore divided into three broad groups of interest: feasibility, tolerability and treatment effects. These results are reported for each intervention category in Tables 1, 2, 3 and 4 described in further detail below. Where adverse events were attributed by authors to the dietary intervention, they have been included under “tolerability”. Where they were reported in relation to their treatment e.g. chemotherapy side effects, they are included under “treatment effects”.

**Table 1:** Ketogenic diet results table

Reference (author, year, country)	Design	Population (No. of participants, age, site/lesion type)	Intervention (DR intervention, corresponding cancer treatment)	Feasibility	Tolerance	Treatment effect
Cohen, 2016, USA [21]	Feasibility RCT	73 randomised, 45 analysed (25 in IG, 20 in CG) Mean age 60.2y (range 31-79y) Recurrent ovarian cancer	KD: 5% CHO, 25% protein, 70% fat over 12 wks. Usual care (24% received concurrent chemotherapy)	62% retention 80% adherence (defined as ∼0.5 mmol/L urinary ketone conc.)	↔ lean body mass between groups ↓total body fat (kg) (32.7 ± 3.1 vs 41.2 ± 4.4) android fat (kg) (2.8 ± 0.4 vs 3.6 ± 0.5), and visceral fat (g) (975 ± 150.9 vs 1024 ± 175.6) (*p* < 0.05) in IG	↓insulin (μU/mL) in IG (6.7 ± 0.9 vs 12.1 ± 1.5, *p* < 0.01) ↔ glucose ↓C-peptide in IG (2.0 ± 0.3 vs 3.0 ± 0.3, *P* < 0.01) ↔ IGF-I or IGFBP-1 ↑ physical component scores in IG (45 vs 40 *p* = 0.04) ↔ mental component score ↑cravings for salt (*p* = 0.03), and ↓cravings for starchy foods (*p* = 0.03) and fast food fats (*p* = 0.04) in IG ↔ cravings for high-fat foods or sweets
Anderson 2016, USA [22]	Phase 1 trial with single assignment	9 Age NR Stage 3-4b head and neck squamous cell carcinoma	4:1* KD fed by PEG followed by oral intake for 5 wks. Concurrent platinum chemo-radiotherapy	33% retention Ppts who discontinued completed a median of 6 days (range 0–8 days) on KD Trial terminated early (intended sample size 14)	6 discontinued: additional stress (*n* = 1), grade 2/3 nausea (*n* = 3), grade 3 fatigue (*n* = 1), grade 4 hyperuricemia (*n* = 1) 2 SAEs: hyperuricemia, pancreatitis	4 SAEs: parotiditis, nausea, vomiting, neutropenic fever ↑Ketones in compliant ppts (median 24 days, range 19–25 days) ↑BHB levels in compliant ppts (median 5 wks, range 4-5wks) ↔ lipid panel test at 3wks ↑ Serum oxidative stress markers with increasing days on KD
Renda 2015 and Dardis, 2017, USA [23]	Phase 1/2 trial with single assignment	14 Mean age 45y (range 37-63y) Brain cancer	4:1 KD* for 8 wks during concurrent radiation and chemotherapy, followed by a 1:1 diet during adjuvant temozolomide chemotherapy	47% recruitment 14% stopped due to tolerability Trial terminated early (intended sample size 40)	No weight loss > 10% of baseline (NB - only reported in preliminary results from 6 ppts)	29% reported nausea
Rieger 2010 and Rieger, [24], Germany [25]	Pilot study with single assignment	20 Mean age 55y (range 30-72y) Brain cancer	KD: < 60 g/day CHO consumed with 500 ml highly fermented yoghurt drinks and 2 plant oils daily. Followed diet for 6–8 wks alone and for a further 6–8 wks either alone or during salvage chemotherapy (*n* = 8)	15% discontinued after 2–3 wks (diet negatively affecting QoL)	↓ body weight (− 2.2%) at 6–8 wks No SAEs attributable to diet	No grade 3 AEs 12 out of 13 evaluable ppts achieved ketosis (73% of urine samples had detectable ketosis) ↔ blood glucose and HbA1c at 6–8 wks
Zahra, 2017, USA [26]	Phase 1 trial with single assignment	9 Age range 51-83y Non small cell lung cancer (*n* = 7) Pancreatic cancer (*n* = 2)	4:1 KD: 90% fat, 8% protein,2% CHO (KetoCal powder + food provided). KD 2 days prior to chemo-radiotherapy until end of treatment (6wks for lung and 5wks for pancreatic)	71% withdrawal in lung cancer ppts: Difficulty complying (*n* = 4), grade 4 hyperuricemia (*n* = 1) 50% withdrawal in pancreatic cancer ppts: Grade 3 dehydration (*n* = 1) Average time on diet: 16.9 days (0–42) for lung and 21 days (8–34) for pancreatic cancer ppts	↓body weight in lung (−6%) and pancreatic (−9.75%) cancer ppts	Grade 3/4 nausea (*n* = 1), dehydration (*n* = 1), esophagitis (*n* = 1) Ketosis achieved in 89% Ketosis maintained in 33% ↔ blood glucose ↑ median plasma protein carbonyl content (nmol/mg) from pre- to post- diet (1.0 vs ≈ 1.4, *p* < 0.05)
Artzi, 2017, Israel [27]	Non randomised trial	9 (5 in IG, 4 in CG) IG: mean age 51y (range 37–69y) CG: mean age 46y (range 27–64y) Brain cancer	4:1 KD using KetoCal® formula for 2–31 months Bevacizumab, temozolomide or rindopepimut	40% adherence (self-report and urine ketones; ppt considered adherent when ketone level was > 2 urine ketosis)	80% tolerance (tolerability criteria not defined)	Evidence of ketone bodies within the brain found in 67% of cases and 0% of controls
Champ, 2014, USA [28]	Retrospective case control study	53 (6 cases, 47 controls) Mean age 54y (range 34-62y) Grade 3–4 glioblastoma	“Patient driven KD” – CHO levels below 50 g/day or 30 g/day if ketosis not reached Chemo-radiotherapy or adjuvant chemotherapy	NR	Grade 1 constipation (*n* = 2) Grade 2 fatigue (*n* = 1) No grade 3 toxicity	Confirmed ketosis in all cases ↓mean glucose in cases from 142.5 mg/dl (range 82–181 mg/dl) to 84 mg/dl (range 76–93 mg/dl) (*p* = 0.02)
Klement, 2016, Germany [29]	Case series	6 Mean age 60y (range 40-74y) Breast (*n* = 1), prostate (*n* = 1), rectal (*n* = 3) and lung (*n* = 1) cancer	KD: 80% fat and < 50 g/day CHO during treatment (mean 48.2 days, range 32–73 days) Radiotherapy or chemo-radiotherapy	100% adherence rate to < 50 g/day CHO consumption Average energy from fat 73% Low BHB and high glucose in some ppts self-reporting as adherent	KD more satiating than previous diet (self report) General subjective feeling on diet rated as “good” 100% reported they would continue with a low CHO/KD after RT ↓ weight (kg/wk) in 33% ↓FM in 50% ↔ absolute FFM ↑ FFM relative to body weight in 50%	↑ (worsening) symptom scores for fatigue, nausea/vomiting, appetite loss, diarrhoea ↑ in BHB ↔ glucose ↔ global health status and total functional scores
Attar [30] and 2016, USA [31]	Retrospective review	13 Age range 23-72y (mean NR) Recurrent brain cancer	Modified Atkins Diet: up to 60 g/day carbohydrate (2–5% total calories) from 1 to 21 months 9 on chemotherapy	85% adherence (range 1–21 months)	2 discontinued: weight loss (*n* = 1), inconvenience (*n* = 1) 1 SAE: renal calculus at 11 months	100% achieved ketosis
Randazzo, 2015, USA [32]	Retrospective data registry review	596 (81 cases, 515 controls) Mean age 49.6y (range NR) Brain cancer	Self-administered “special diets” including KD, Low CHO, vegetarian/vegan Usual care	NR	NR	NR – data not stratified by diet type

*4:1 KD: A ketogenic diet consisting of 80% energy intake from fat

↑ = increase/higher

↓ = reduction/lower

↔ = no change/no difference

≈ = approximate

Where absolute figures were provided, %s have been calculated to aid comparison

*Abbreviations: AEs* Adverse Events, *BHB* Beta-hydroxybutyrate, *CG* Control Group, *CHO* Carbohydrate, *DLT* Dose Limiting Toxicities, *DR* Dietary Restriction, *FM* Fat Mass, *FFM* Fat Free Mass, HbA1c Glycated Haemoglobin, *HPD* highest posterior density interval, *IG* Intervention Group, *IGF* Insulin-like Growth Factor, *IGFBP* Insulin-like Growth Factor Binding Protein, NR Not Reported, PEG Percutaneous Endoscopic Gastrostomy, Ppt Participant, QoL Quality of Life, *RCT* Randomised Controlled Trial, SAEs Serious Adverse Events

**Table 2:** Protein restriction results

Reference (author, year)	Design	Population (No. of participants, age, site/lesion type)	Intervention (DR intervention, corresponding cancer treatment)	Feasibility	Tolerance	Treatment effect
Eitan, 2017, USA [33]	RCT	38 (19 IG, 19 CG) Mean age 59.26 ± 7.5y Prostate cancer	Protein restricted diet (0.8 g protein kg − 1 lean body mass) Awaiting surgery (43 ± 11 days on diet)	NR	NR	↔ EV size in either arm ↑ Levels of EV-associated LeR ↑ Y/S IRS1 ratio in neuronal-enriched EVs in IG vs CG ↓Body weight (kg) (− 2.62 ± 2.18 *p* = < 0.0001), FM (kg) (− 1.37 ± 1.55 p = 0.001), and BMI (− 0.76 ± 0.75 *p* = < 0.0001) in IG
Durando, 2008, France [34]	Phase 1 clinical trial with single allocation	10 Median 68y (range 35-76y) 9 metastatic melanoma, 1 recurrent glioma	MET-free diet ranging from 1 to 4 days over 4 cycles of cystemustine chemotherapy	Ppts consumed 72.4% ± 31.5% of the MET-free diet administered	↔ BMI, plasma albumin or NRI	↓MET conc., optimal depletion obtained on day 1 (− 40.7 ± 36.9% *p* < 0.05) Nitrogen balance (g/24 h) stable and negative during MET-free diet (− 2.24 ± 3.16) ↓Daily 3MH:creatinin ratio from 29.9 ± 14.9 × 10–3 at D0 to 15.9 ± 4.9 × 10–3 at D4 (*p* < 0.05) Grade 3 thrombocytopenia (33%), neutropenia (33%) and leucopenia (20%)
Durando, 2010, France [35]	Feasibility study with single arm assignment	11 Median age 70y (range 48-78y) Metastatic colorectal cancer	MET-free diet for 3 days over 3 cycles of FOLFOX chemotherapy	Patients consumed 92.5% ± 21.8% of the MET-free diet administered	↔ BMI: 24.6 ± 3.vs 24.3 ± 2.9 (*p* = 0.12) ↔ plasma albumin: 36.0 ± 8.6 vs 36.7 ± 8.3 g/l (*p* = 0.76)	↓MET concentrations. Day 1: − 58.1 ± 19.1%. Day 3: − 43.3% ± 13.9% Grade 3 neutropenia without fever (9%) No grade 3–4 non-haematological toxicities
Thivat, 2007, France [36]	Phase 1 trial with single arm assignment	6 Age NR 1 recurrent glioma, 5 metastatic melanoma	MET-free diet ranging from 1 to 4 days over 4 cycles of cystemustine chemotherapy	NR	NR	↓Plasma MET of 48.5 ± 4% from 23.1 ± 1.6 μg/L to 11.3 ± 0.7 μg/L (*p* = 0.00002) Grade 3–4 thrombocytopenia (33%), neutropenia (33%) and leucopenia (33%) ↓ MGMT activity (fmol/mg of protein) 553 ± 90 to 413 ± 59 (*p* = 0.029). Mean ↓ of 36 ± 8% No effect of duration of diet on MGMT activity after treatment
Thivat, 2009, France [37]	Phase 2 trial with single arm assignment	22 Median age 62y (range 35-76y) 20 melanoma, 2 glioma	1 day MET-Free over 4 cycles cystemustine chemotherapy	Patients consumed 78 ± 27% of the MET-free diet administered	↔ body weight (kg) (68.8 ± 11.5 vs. 67.8 ± 11.4, *p* = 0.11), plasma albumin (g/l) (from 37.8 ± 5.6 to 36.6 ± 6.8, *p* = 0.09) or prealbumin (g/l) (from 0.25 ± 0.1 to 0.23 ± 0.1, *p* = 0.32)	↓Plasma MET of 53.1 ± 21.8% after 4 h Grade 3–4 thrombocytopenia (36%), neutropenia (27%) and leucopenia (27%)

↑ = increase

↓ = reduction

↔ = no change

≈ = approximate

Where absolute figures were provided, %s have been calculated to aid comparison

*Abbreviations: BMI* Body Mass Index, *CG* Control Group, *DR* Dietary Restriction, *EV* Extracellular Vesicles, *FM* Fat Mass, *IG* Intervention Group, *Y/S IRS1* Insulin Receptor Substrate, *LeR* Leptin receptor, *MET* Methionine, *MGMT* DNA repair protein O(6)-methylguanine-DNA methyltransferase, *NR* Not Reported, *NRI* Nutrition Risk Index, *Ppts* Participants, *RCT* Randomised Controlled Trial, *3MH* Urinary 3-methylhistidine

**Table 3:** Fasting results

Reference (author, year)	Design	Population (no. of participants, cancer site, treatment)	Intervention (DR intervention, corresponding cancer treatment)	Feasibility	Tolerance	Treatment effect
De Groot 2013 and 2015, Netherlands [38, 39]	Pilot RCT	13 (7 IG, 6 CG) IG: Median age 51y (range 47-64y) CG: Median age 52y (range 44-69y) Stage 2–3 breast cancer	48 h fast (24 h before until 24 h after start of chemotherapy) 3 weekly (neo) adjuvant TAC-chemotherapy	15% withdrawal	NR	↑ median blood glucose (mmol/L); IG: 5.2 to 6.8 (*p* = 0.042), CG: 4.8 to 7.0 (*p* = 0.043) ↓ mean IGF-1 (nmol/L) of 17% in IG (23.7 to 19.6, *p* = 0.012), ↔ CG ↔ median insulin (mU/L) in IG, ↑ in CG group**:** 2.0 to 16.0 (*p* = 0.043) ↔ TSH (mU/L) in IG: 1.49 to 0.42, ↓ in CG: 1.38 to 0.61 (*p* = 0.034) ↔ in IGF-BP3 or FT4 ↑ erythrocytes in IG (Day 7: *p* = 0.007, 95% CI 0.106–0.638; Day 21: *p* = 0.002, 95% CI 0.121–0.506) ↑ thrombocytes in IG (*p* = 0.00007, 95% CI 38.7–104) at day 7 ↔ leukocytes or neutrophils ↔ self-report side effects
Dorff, 2016 and Quinn, 2013, USA [40]	Dose escalation	20 Median age 61y (range 31–75y) Any cancer	3 cohorts fasted before chemotherapy for 24, 48 and 72 h (divided as 48 pre-chemo and 24 post-chemo) Platinum based chemotherapy	Adherence: 24 h fast: 67%, 48 h fast: 83%, 72 h fast 57%	Grade 1/2 fatigue, headache, dizziness, hypoglycaemia, weight loss, hyponatremia and hypotension No grade 3/4 fasting-related toxicities 5% failed to regain 25% of weight lost	↓ IGF1. 24 h fast: Cycle 1: − 30% (− 12 to − 44%) Cycle 2: − 31% (− 45% to − 13%) 48 h fast: Cycle 1: − 33% (− 45% to − 18%) Cycle 2: − 20% (− 37 to 1%) 72 h fast: Cycle 1: − 8% (− 24 to 13%) Cycle 2: 16% (− 5 to − 42%) ↔ glucose ↓ mean insulin. 24 h fast: − 56%. 48 h fast: − 27%. 72 h fast: − 42% at 48 h (data at 72 h NR) ↓ DNA damage in 48 h and 72 h, but not 24 h fast ↓ nausea. 24 h fast: 100%, 48 h fast: 87%, 72 h fast: 43% (*p* = 0.019) ↓ vomiting. 24 h fast: 83%, 48 h fast: 43%, 72 h fast: 0% (*p* = 0.003) ↔ neutropenia. 24 h fast: 67%, 48 h fast: 14%, 72 h fast: 29% (*p* = 0.17)
Mas, 2017, France [41]	Qualitative	15 Age NR Breast cancer	Self-administered fast concurrent to chemotherapy	Main motivation to limit chemotherapy side effects Effect of fasting on tumour was not a motivation (patients felt cancer-free following surgery) Offered a chance for ppts to take an active role in treatment	13% reported AEs which stopped them fasting	Fasting was a positive experience that reduced the side effects of chemotherapy and reinforced self-esteem
Safdie, 2009 and [42], USA [43]	Case series	10 Median age 61y (range 44-78y) Breast (*n* = 4), prostate (*n* = 2), ovarian (*n* = 1), uterine (*n* = 1), lung (*n* = 1), oesophageal (*n* = 1) cancer	Self-administered fast ranging from 48 to 140 h prior to and/or 5–56 h following chemotherapy	NR	Low grade dizziness, hunger, and headaches reported No grade 3/4 toxicities Weight loss recovered in “most” patients	↓ in fatigue (*p* < 0.001), weakness (*p* < 0.00193) and GI side effects (absent) in 46 reported cycles with fasting compared with 18 ad-libitum cycles

↑ = increase/higher

↓ = reduction/lower

↔ = no change/no difference

Where absolute figures were provided, %s have been calculated to aid comparison

*Abbreviations: AEs* Adverse Events, *CG* Control Group, *CHO* Carbohydrate, *DR* Dietary Restriction, *FT4* thyroxine, *GI*, gastrointestinal, *IG* Intervention Group, *IGF* Insulin-like Growth Factor, *IGFBP* Insulin-like Growth Factor Binding Protein, *NR* Not Reported, *RCT* Randomised Controlled Trial, *SAEs* Serious Adverse Events, *TSH* Thyroid Stimulating Hormone

**Table 4:** Combined intervention results

Reference (author, year)	Design	Population (No. of participants, age, site/lesion type)	Intervention (DR intervention, corresponding cancer treatment)	Feasibility	Tolerance	Treatment effect
Freedland, 2016, USA [44]	RCT	40 (19 IG, 21 CG) Age NR Prostate cancer	Low CHO diet (< 20 g/day) combined with moderate physical activity increased by 30 min/day for 5 days/wk. Concurrent to ADT	81% retention	Mild headaches main side effect	↓ HOMA by 19% in IG compared to 7% in CG (*p* = 0.127) at 3 m ↓ weight (kg) of 9.3 in IG compared to ↑ of 1.3 in CG (*p* < 0.001) at 6 m ↓ FM of 16.2% in IG compared to ↑ of 11.0% in CG (*p* = 0.002) at 6 m ↑ bone mineral content of 0.1% in IG compared to ↓2.3% in CG (*p* = 0.025) at 6 m ↓ PSA 99% in both groups (*p* = 0.37)
Reinwald, 2015 and Branca, [45], Italy [46]	Case report	1 Age 66y Breast cancer	An isocaloric KD: special amino acid formula combined with probiotic yoghurt containing vitD binding protein macrophage activating factors and injections of vitD, oleic acid and vitD binding protein 3 weeks prior to surgery	NR	NR	Change in gene expression to HER2 -ve. Increase in progesterone expression (20 vs < 1%) No invasion of blood or lymph vessels around the tumour ER and Ki-67 markers were unchanged
Iyikesici, 2017, Turkey [47]	Case report	1 Age 29y Triple negative breast cancer	Chemotherapy administered after a 12 h fast followed by 5–10 units of insulin. Patient also consumed a KD for duration of treatment	Patient adhered to KD (urinary ketones present at each visit)	NR	Pathological complete response
Zuccoli, 2010, Italy [48]	Case report	1 Age 65y Brain cancer	Self-administered post-operative fast followed by a calorie restricted KD with chemo-radiotherapy. KD: 600ckal/day using Keto-Cal® 4:1 supplemented with multivitamins. After approx. 2 months on restricted KD, patient switched to a calorie restricted non-KD (600 kcal/day) for 5 months.	NR	Karnofsky performance status: 100% during diet Hyperuricemia on restricted KD so patient was switched to a non-KD calorie restricted diet. Hypoproteinemia on restricted diet, resolved by increasing dietary protein to 7 g/day for 1 month. ↓ bodyweight (− 9%) after fast and − 22% after restricted diet	↓ blood glucose: −50% after fast and − 53.3% after restricted diet ↑ ketones: from 0 (baseline) to 2.5 mmol/L after fast and after restricted diet

↑ = increase

↓ = reduction

Where absolute figures were provided, %s have been calculated to aid comparison

*Abbreviations: ADT* Androgen Deprivation Therapy, *CG* Control Group, *CHO* Carbohydrate, *DR* Dietary Restriction, *ER* Estrogen receptor, *FM* Fat Mass, *HER-2* Human Epidermal Growth Factor Receptor 2, *HOMA* Homeostatic model assessment, *IG* Intervention Group, *KD* Ketogenic Diet, *m* months, *NR* Not Reported, *PSA* Prostate-Specific Antigen, *RCT* Randomised Controlled Trial, *VitD* Vitamin D

### Ketogenic diets

Ten studies of KDs that were conducted alongside treatment for cancer were identified: one randomised controlled trial (RCT) [21], four single arm trials [22, 23, 25, 26], one non-randomised, parallel design trial [27] one case control study [28], one case series [29] and two retrospective reviews [31, 32]. Four of the studies also included participants who were not on any active treatment at the time of the DR [21, 25, 31, 32]. However, as they reported on outcomes of interest relevant to our research question (e.g., adherence) they were included in the review. The majority of KD studies were in people with brain cancer (*n* = 6) and the most common form of diet was a 4:1 ratio KD (*n* = 5). The results are summarised in Table 1.

Feasibility results were varied. Of the six interventional studies, two were terminated early due to poor accrual and adherence [22, 23]. In the remaining four, the proportion of non-completers ranged from 15 to 71%. Adherence was reported in two of the interventional studies and was 40% [27] in one study and 80% [21] in the other. However, although both studies used urinary ketones different cut-offs were used to assess adherence.

Weight loss, adverse events and reasons for discontinuation of diet were the main tolerability outcomes reported. In general, weight loss was not a cause for concern on the KDs used, with loss remaining below 10% of initial body weight in the majority of participants. Two trials also broke down weight lost into loss of fat mass and fat free mass. Both found that in spite of weight loss, fat free mass was preserved [21, 29]. Reports of grade 3/4 adverse events were rare.

Intervention effects reported included markers of metabolism such as ketones, glucose and insulin, quality of life and treatment- related adverse events. Of the seven studies that reported on ketones or βeta-hydroxybutyrate specifically (a common ketone), all reported ketosis or an increase in ketones in those on the KD. However, this was not always linked with a corresponding reduction in blood glucose [21, 25, 26, 29]. Champ et al is the exception which found a reduction in blood glucose on KD during radiotherapy, even though participants received steroidal treatment which is known to increase blood glucose [28]. Four studies reported on quality of life [21, 25, 29, 32] with one finding evidence of positive effects [21], one finding negative effects [25] and one finding no effect [29]. We were unable to extract results from the fourth study as they were not stratified by diet type [32].

### Protein restriction

Five studies of protein restriction were identified, of which four were specifically methionine (MET)-restricted (Table 2). One study was an RCT in people with prostate cancer [33] while the remaining four were clinical trials with single arm allocation [34–37] including people with melanoma, glioma and colorectal cancer. One of the single arm trials was a phase 1 trial [36] which was followed by a phase 2 trial [37].

MET free diets were delivered as oral powders which participants consumed as drinks. Three of the four MET-free diet studies reported on the mean adherence to the diet which ranged from 72.4 to 92.5% [34, 35, 37]. Feasibility findings were not reported in the RCT of a protein restricted diet [33].

Tolerability was reported in three trials of the MET restriction. There were no changes in markers of nutritional status (body weight, albumin or pre-albumin) associated with the MET-free diet [34, 35, 37]. In the protein restricted diet trial, the intervention group lost weight, but this was an aim of the trial which recruited overweight participants [33].

The main outcome of interest within the MET restriction studies was blood MET concentration. All four trials of MET-free diet resulted in a reduction in mean plasma MET concentrations (reductions ranged from 40.7 to 53.1%) which authors reported as successful reduction rates [34–37]. Outcomes of interest in the total protein restriction trial were cellular effects of the diet, specifically the effect of the diet on molecular mediators in extracellular vesicles. They found that the diet increased the levels of extracellular vesicle-associated leptin receptors and a higher Y/S Insulin receptor substrate-1 ratio in the protein restricted group, indicating improved leptin and insulin sensitivity [33].

### Fasting

Four studies of fasting were identified, and all were conducted at the time of chemotherapy: One pilot RCT [38], one dose escalating study [40], one qualitative study [41] and a case series report [43] (Table 3). Each study utilised a different fasting protocol. Self-administered fasts ranged from 48 to 140 h prior to and/or 5–56 h following chemotherapy. Per-protocol fasts ranged from 24 h prior to chemotherapy to 72 h, divided as 48 h prior to chemotherapy and 24 h post-chemotherapy. Each study also included a different clinical population, with varying cancer types.

Feasibility findings were reported in both interventional studies with the pilot RCT reporting a 15% (*n* = 2) withdrawal rate [38]. Within the dose escalation study, authors reported 67% compliance in the 24 h fast, 83% in the 48 h fast and 57% in the 72 h fast. However, they also note that, although self-reported compliance was high in the 72 h fast, it may have been subject to poorer compliance given that the IGF analysis showed lower than expected reductions in IGF1 at 72 h [40].

Tolerability of the fast was not discussed in the RCT. However, no grade 3/4 toxicities were reported among participants in the dose-escalating or qualitative studies [40, 43]. Grade 1/2 toxicities are listed in Table 3 and included dizziness, hunger, headaches and weight loss. Among the participants in the qualitative study, 13% (*n* = 2) reported experiencing adverse events which stopped them following their self-administered fast [41]. In the case series, weight loss was reported to resolve in “most” participants following introduction of normal feeding [43]. Only 1 participant in the dose escalation study did not regain at least 25% of body weight last during the fast between cycles and was unable to continue with the second fast, as per the trial protocol [40].

The intervention effects of interest within the fasting literature focus on biological markers of metabolism and chemotherapy toxicities. Both interventional studies found a reduction in IGF1 associated with fasting, however the levels were varied depending on the trial and length of the fast. Reductions ranged from 17.3% after 24 h of the 48 h fast [38] to 33% after the 48 h fast [40]. Despite fasting, neither interventional study found a reduction in glucose, with glucose increasing after 24 h in the RCT [38] and no changes evident in the dose escalation study [40]. Study authors suggested the use of steroidal treatment among study participants as a potential reason for the lack of glucose reduction during fasting. The two observational studies found evidence of decreased side effects from chemotherapy. This was self-reported in the qualitative study [41] (side effects that were reduced were not specified) while the case series report found a reduction in fatigue, weakness and gastrointestinal side effects in cycles completed alongside a fast when compared to cycles where cases ate ad-libitum [43]. These findings were similar in the dose escalating study which found a trend for reduced nausea and vomiting in longer fasts [40] but were not seen in the pilot RCT which found no differences in self-reported AEs between groups [38].

### Combined interventions

Four studies of combined interventions were identified and are summarised in Table 4: One RCT [44] and three case reports [46–48]. All combined some form of ketogenic or low carbohydrate diet with additional interventional aspects such as increased physical activity in the RCT [44], or additional dietary changes [46–48]. As the diets were delivered in combination with other components and the majority are based on single patient case reports, interpretation is limited. However, the RCT reported a high retention rate of 81% and found that the main side effect associated with the low carbohydrate and increased physical activity intervention was mild headaches [44].

### Ongoing/planned trials

The clinicaltrials.gov and ISRCTN databases were searched on 10th December 2018 for studies that were registered as ongoing or planned. This search identified: 13 trials of KD, one trial of a KD combined with short term fasting, one trial of short term fasting, five trials of fasting-mimicking diets and two trials of intermittent fasting. These are summarised in Table 5. This search indicates that the KD continues to be the most researched form of restriction (*n* = 13) and the majority of these studies are in people with brain cancer (*n* = 8). Although there are an increasing number of KD RCTs identified (*n* = 5), three specifically identify as pilot/feasibility studies, and all have small target sample sizes (range = 12–60). An increased interest in other forms of fasting such as intermittent fasting (*n* = 2) and fasting-mimicking diets (*n* = 5) is also evident. Fasting RCT target sample sizes range from 30 to 250.

**Table 5:** Planned/ongoing trials registered in clinicaltrials.gov and ISRCTN databases

Trial Registration	Trial design	Participants (target recruitment number and type of cancer)	Primary outcomes	Planned start and end dates
Ketogenic Diets				
NCT03285152	Feasibility RCT	30 Endometrial	No. of patients that complete the study	Aug 2017- Aug 2019
NCT02983942	Pilot RCT	50 Primary Central Nervous System Lymphoma	No. and incidence of treatment related AEs	Jan 2017 – Dec 2019
SRCTN71665562	Pilot RCT	12 Glioblastoma	Retention rate	Jul 2016 – June 2019
NCT02302235	RCT	42 Glioblastoma	1. Survival time 2. Time to progression (MRI assessed) 3. Incidence of AEs	Feb 2014 – Dec 2018
NCT01754350	RCT	50 Glioblastoma	Progression-free-survival rates 6 months after reirradiation	May 2013 – June 2018
NCT03278249	Feasibility trial (single arm)	30 Glioblastoma	Ketosis (measured by serum BHB)	Sep 2017 – Jan 2021
NCT01865162	Pilot trial (single arm)	6 Glioblastoma	Safety evaluation	Jan 2013 – Jan 2019
NCT01535911	Pilot trial (single arm)	16 Glioblastoma	Brain tumour size response (MRI assessed)	Apr 2012 – June 2019
NCT03451799	Clinical trial (single arm)	Glioblastoma	Safety assessed by weight loss and AEs	Apr 2018 – Sep 2020
NCT02964806	Crossover trial	30 Pancreaticobiliary Cancer	Diet intake rate	Nov 2016 – Oct 2017
NCT02939378	Non-randomised trial (2 arms)	60 Glioblastoma	No. of participants with AEs	Oct 2016 – Dec 2018
NCT03535701	Non-randomised trial (2 arms)	15 Breast	1. Adherence 2. Change in psychosocial measures 3. Change in physiologic outcomes	Oct 2017 – Aug 2019
NCT03194516	Observational	12 Prostate	Weight loss at 8 weeks	June 2017 – May 2021
Short-term Fast				
SRCTN17994717	Feasibility RCT	30 Colorectal	Adherence, recruitment, retention and data completion rates Acceptability and tolerability	Oct 2017 – Apr 2021
Fasting Mimicking Diets				
NCT02126449	RCT	250 Breast	1. Rate of grade 3/4 toxicity 2. Rate of pCR	Feb 2014 – Dec 2019
NCT03700437	RCT	40 Lung	Effect of diet on circulating tumour cells	Oct 2018 – Dec 2020
NCT03340935	Feasibility trial (single arm)	85 Any cancer	AEs	Feb 2017 – June 2018
NCT03595540	Pilot trial (single arm)	60 Breast and colorectal	1. % diet consumed 2. Diet related AEs	Nov 2017 – Sep 2020
NCT03454282	Clinical trial (single arm)	100 Breast and melanoma	Change in peripheral blood mononuclear cell	May 2018 – Dec 2020
Intermittent Fasts				
NCT03162289	RCT	150 Breast	Change in FACT-G Quality of Life score	May 2017 – May 2022
NCT02710721	RCT	60 Prostate	Change in FACT-P Quality of Life score	April 2016 – Dec 2019
Ketogenic Diet combined with Short-Term Fast				
NCT02286167	Clinical trial (single arm)	25 Glioblastoma Multiforme	Dietary adherence rates	Nov 2014 – April 2019

*Abbreviations: AE* Adverse Events, *BHB* Beta-hydroxybutyrate, *MRI* Magnetic Resonance Imaging, *pCR* Pathological Complete Response, *RCT* Randomised Controlled Trial

## Discussion

### Main findings

Few studies have been published on DR during treatment for cancer to date, particularly when the data are stratified by restriction type. More studies are currently in progress and due to complete recruitment within the next 3 years, which identifies DR as a research area of growing interest. However, most ongoing trials are early stage studies with small sample sizes. These may allow us to further understand the feasibility of conducting such studies but will not enable conclusions to be drawn about the efficacy of these interventions. Large studies with long-term outcomes are needed to definitively address these questions.

Our findings show that the most commonly studied form of dietary restriction is the KD. As with the previous review of KD in adults with cancer not specifically receiving treatment for cancer, we found the 4:1 diet to be the most common form being used in conjunction with treatment [17]. The previous review concluded that adherence rates were low, and our review confirms the potential issues surrounding adherence when using KD alongside treatment for cancer. Adherence results were varied, with different definitions of adherence and tolerability used, making comparisons of adherence to the different forms of the KD difficult. This, in combination with the early termination of two of the KD trials, suggest that further research into improving acceptability of KDs may be warranted. For example, there could be the potential for improved adherence and retention in KD studies with lower ratios of fat:carbohydrate than the 4:1 diet whilst still achieving favourable metabolic changes [26]. Furthermore, as most studies of KD reported few issues with tolerability and weight loss, it is possible that there could be issues with palatability or sustainable behavioural change.

The results of protein restriction research suggested that MET-free diets were adhered to well with limited tolerability issues. However, the diets were provided over a short amount of time as oral solutions. It is less clear how well general protein restriction as part of a low protein meal-based diet is adhered to, as only a single trial of overall protein restriction has been conducted, which did not report feasibility outcomes.

Very few studies of fasting have been conducted. Overall, the studies to date have found that participants are able to follow short-term fasts, although length of interventions varied, and it is unclear whether longer fasts have lower adherence. As with the other dietary restriction methods, adverse events related to fasting did not appear to affect adherence in the majority of studies, with the exception of the qualitative study [41]. This may be because participants in that study were self-administering the fast and not receiving clinical support. As with the research on KDs, fasting appears to result in a reduction in IGFs. However, it remains unclear whether it also results in a decrease in blood glucose. This may be due to steroidal treatment received alongside chemotherapy, which is known to increase blood glucose levels. One interventional and two observational studies found some evidence of reduced toxicities in fasted participants, however the evidence is limited by the small number of trials and small sample sizes included.

### Future research

Larger, adequately powered RCTs will be required in order to study the efficacy of each DR intervention type to reduce treatment side effects or improve outcomes. Within KD research, further exploration of issues associated with adherence is warranted if larger trials are to test this intervention. There is a current lack of in-depth qualitative work conducted in this area, which may help in exploring the reasons for non-compliance in trials, especially if tolerability is high.

While research into MET-free diets suggest that trials of this intervention are feasible, definitive RCTs with larger sample sizes are required to ascertain whether these diets result in reduced treatment side effects or improved outcomes. Further research into adherence to and tolerability of general protein restricted diets is required in order to understand the feasibility of conducting this form of intervention alongside treatment of cancer. It is also not clear whether this diet could be introduced to people with normal weight without resulting in significant weight loss, as the only trial to date was in people who were overweight.

Conflicting findings regarding blood glucose levels suggest further research into the effect of dietary restriction on this marker is required. Attention should also be paid to the use of steroidal treatment alongside chemotherapy, to investigate whether increased blood glucose seen with these drugs inhibits the potentially protective effect of dietary restriction. In a current study of fasting-mimicking diets the investigators have chosen to omit dexamethasone treatment [39]. However, on a pragmatic level, it would also be of interest to explore whether IGF reduction alone is able to induce metabolic changes that would be sufficient to achieve a reduction in toxicity, even in the presence of dexamethasone. Particularly as two observational and one interventional study found some evidence for reduced side effects when chemotherapy was provided as standard. Reporting on the type of weight-loss resulting from the fast would also be of interest, to ascertain whether fat is lost while lean muscle mass is retained, as has been the case in KDs.

### Strengths and limitations

While some aspects of dietary restriction have been reviewed previously [16, 17], this scoping review employed a systematic search of the literature on the different forms of dietary restriction during treatment for cancer, to collate the research to date. Although every effort was made in the search to identify all relevant texts, it is possible that some studies of DR during treatment for cancer have been missed.

In order to acknowledge the emerging nature of DR research, a scoping review process was followed, which included data from observational and single-arm studies. This allowed us to consider the breadth of previous research in an emerging field, helping to inform future studies. However, this also means that the quality of studies has not been assessed against the standards commonly used in systematic reviews of RCTs. This approach has allowed us to summarise the emerging research on DR in cancer treatment and highlights some issues that should be considered when designing further studies in this area.

### Conclusion

DR regimes are a potential tool to help reduce the toxicities associated with cancer treatment. However, the limited number of studies to date have had small samples and have not been designed to specifically test the efficacy of these interventions. DR is, however, a growing research area with further trials being conducted. Definitive RCTs are required to assess the efficacy of DR during cancer treatment on reducing treatment related toxicities or improving treatment outcomes. This scoping review has highlighted the potential problem of adherence issues and as such suggests further research into improving dietary compliance is conducted before larger efficacy trials are conducted. Further research into the effect of DR interventions on cellular metabolism when used in combination with treatment is also warranted.

## Additional file

Additional file 1: Search terms used in the Medline database search. (DOCX 13 kb)

## Data Availability

Data is currently only accessible to members of the study research team but may also be made available on reasonable request by contacting the corresponding author.

## Abbreviations

BRC: Biomedical Research Centre
DR: Dietary Restriction
IGF: Insulin-like Growth Factor
ISRCTN: International Standard Randomised Controlled Trial Number registry
KD: Ketogenic Diet
MET: Methionine
NIHR: National Institute for Health Research
RCT: Randomised controlled trial
