# Supplementary material for: Dietary restriction during the treatment of cancer: results of a systematic scoping review
Source: BMC Cancer. 2019 Aug 15;19:811. doi: 10.1186/s12885-019-5931-7 (PMC6694513; doi:10.1186/s12885-019-5931-7)
Supplement: Supplementary file 1 — Search terms used in the Medline database search. (DOCX 13 kb) [file 12885_2019_5931_MOESM1_ESM.docx]

Additional file 1 – search terms used in the Medline database search

| 1. Fasting/ |  |
| --- | --- |
| 2. (diet* adj2 restrict*).tw,kf. |  |
| 3. (calorie* adj2 restrict*).tw,kf. |  |
| 4. (intermittent* adj2 fast*).tw,kf. |  |
| 5. Starvation/ |  |
| 6. ketogenic diet.tw,kf. |  |
| 7. exp Ketone Bodies/ |  |
| 8. ((protein or carbohydrate) adj2 restrict*).tw,kf. |  |
| 9. "atkins diet".tw,kf. |  |
| 10. 1 or 2 or 3 or 4 or 5 or 6 or 7 or 8 or 9 |  |
| 11. Neoplasms/ |  |
| 12. cancer*.tw,kf. |  |
| 13. carcinoma*.tw,kf. |  |
| 14. tumor*.tw,kf. |  |
| 15. tumour*.tw,kf. |  |
| 16. sarcoma*.tw,kf. |  |
| 17. malignan*.tw,kf. |  |
| 18. oncolog*.tw,kf. |  |
| 19. 11 or 12 or 13 or 14 or 15 or 16 or 17 or 18 |  |
| 20. 10 and 19 |  |
| 21. animals/ not humans/ |  |
| 22. exp Animals, Laboratory/ |  |
| 23. exp Animal Experimentation/ |  |
| 24. Models, Animal/ |  |
| 25. Rodentia/ |  |
| 26. (rat* or mouse or mice).ti. |  |
| 27. 21 or 22 or 23 or 24 or 25 or 26 |  |
| 28. 20 not 27 |  |
